# Hybrid transfer learning strategy for cross-subject EEG emotion recognition

**DOI:** 10.3389/fnhum.2023.1280241

**Published:** 2023-11-16

**Authors:** Wei Lu, Haiyan Liu, Hua Ma, Tien-Ping Tan, Lingnan Xia

**Affiliations:** ^1^Henan High-speed Railway Operation and Maintenance Engineering Research Center, Zhengzhou Railway Vocational and Technical College, Zhengzhou, China; ^2^School of Computer Sciences, Universiti Sains Malaysia, Penang, Malaysia; ^3^Zhengzhou Railway Vocational and Technical College, Zhengzhou, China

**Keywords:** affective computing, cross-subject EEG emotion recognition, fine-tuning, domain adaptation, few-shot

## Abstract

Emotion recognition constitutes a pivotal research topic within affective computing, owing to its potential applications across various domains. Currently, emotion recognition methods based on deep learning frameworks utilizing electroencephalogram (EEG) signals have demonstrated effective application and achieved impressive performance. However, in EEG-based emotion recognition, there exists a significant performance drop in cross-subject EEG Emotion recognition due to inter-individual differences among subjects. In order to address this challenge, a hybrid transfer learning strategy is proposed, and the Domain Adaptation with a Few-shot Fine-tuning Network (DFF-Net) is designed for cross-subject EEG emotion recognition. The first step involves the design of a domain adaptive learning module specialized for EEG emotion recognition, known as the Emo-DA module. Following this, the Emo-DA module is utilized to pre-train a model on both the source and target domains. Subsequently, fine-tuning is performed on the target domain specifically for the purpose of cross-subject EEG emotion recognition testing. This comprehensive approach effectively harnesses the attributes of domain adaptation and fine-tuning, resulting in a noteworthy improvement in the accuracy of the model for the challenging task of cross-subject EEG emotion recognition. The proposed DFF-Net surpasses the state-of-the-art methods in the cross-subject EEG emotion recognition task, achieving an average recognition accuracy of 93.37% on the SEED dataset and 82.32% on the SEED-IV dataset.

Emotion recognition has emerged as a crucial research task within the field of affective computing (Cimtay et al., 2020; Doma and Pirouz, 2020; Almarri et al., 2021). Currently, emotion recognition holds significant potential applications in various domains, including the diagnosis of affective disorders, affective brain-computer interfaces (Jia et al., 2020), emotion detection of drivers (Zhou et al., 2023a), and mental workload estimation (Tan et al., 2020; Huang et al., 2023; Wang et al., 2023). Emotion is a response to both internal and external stimuli (Jia et al., 2021a). Therefore, human emotions can generally be detected through two types of signals: non-physiological signals and physiological signals. Non-physiological signals encompass facial expressions, speech, gestures, and more. These signals are relatively easy to detect and provide intuitive emotional responses (Cimtay et al., 2020; Tan et al., 2020). However, non-physiological signals such as facial expressions, speech, and gestures can also be deliberately concealed. In contrast, while physiological signals are less accessible for detection and recognition, they are challenging to deliberately mask. Commonly used physiological signals for emotion recognition include the electrocardiogram (ECG), electromyogram (EMG), and electroencephalogram (EEG). Among these, EEG signals stand out due to their high temporal resolution and abundant information. This makes EEG signals particularly suitable for emotion recognition when compared to other physiological signals (Atkinson and Campos, 2016). Therefore, an increasing number of researchers are delving into emotion recognition studies based on EEG signals (Xing et al., 2019).

In recent years, due to its ability to accurately reflect the genuine emotions of subjects, EEG signals have found widespread application in the field of emotion recognition (Jia et al., 2020; Zhou et al., 2023b). Early EEG-based emotion recognition relied on processes like signal denoising, feature design, and classifier learning. For instance, Wang et al. (2011). introduced the Support Vector Machine (SVM) classifier, and Bahari and Janghorbani (2013). proposed the K-Nearest Neighbors (KNN) classifier, both achieving effective emotion classification. However, traditional machine learning techniques are constrained by intricate feature engineering and selection processes. To overcome these limitations, deep learning techniques have been introduced. The continuous refinement of deep learning algorithms has led to significant achievements in the realm of EEG-based emotion recognition. Notably, CNN models proposed by Chen et al. (2019) and Kwon et al. (2018). have substantially enhanced the accuracy of EEG emotion recognition. Additionally, the application of Transformer models in EEG emotion recognition has garnered attention, as exemplified by the EEG Emotion Transformer (EeT) by Liu et al. (2022). and the Joint Dimensional Attention Transformer (JDAT) by Wang et al. (2021b).

Despite the success of deep learning, in EEG-based emotion recognition, significant individual differences among different subjects pose a challenge. This leads to a noticeable decrease in the performance of deep learning models on cross-subject EEG emotion recognition tasks. To address this concern, researchers have increasingly explored transfer learning techniques. Fine-tuning, as an effective knowledge transfer method, has gained widespread adoption. Li et al. (2018b). incorporated fine-tuning to investigate subject transfer and the extent of knowledge sharing among subjects. Zhang et al. (2023) introduced the Self-Training Maximum Classifier Difference (SMCD) model, utilizing fine-tuning to apply a model trained on the source domain to the target domain. However, fine-tuning primarily involves adapting a pre-trained model to a new task within the target domain. Therefore, there might be certain limitations when transferring knowledge from the pre-trained model to the target task. If there are substantial domain differences between the source and target domains, or if the characteristics of the target task do not align well with the original task of the model, the effectiveness of fine-tuning could be constrained. Researchers have begun exploring the application of domain adaptation in cross-disciplinary EEG emotion recognition. Jin et al. (2017) employed a Domain Adaptation Network (DAN) for knowledge transfer, aiming to alleviate source-target subject disparities and eliminate variability. Li et al. (2019) proposed a Domain Adaptation method that enhances adaptability by minimizing source domain error and aligning latent representations. However, domain adaptation primarily reduces domain differences by learning feature representations between the source domain and the target domain. Therefore, relying solely on domain adaptation may not fully accommodate the characteristics of the target task and the variations in the target domain. Models may not adequately leverage the label information in the target domain, resulting in a decrease in performance. Domain adaptation typically focuses on addressing the disparities between a model's performance in the source domain and the target domain. Fine-tuning further enhances the performance of the model in the target domain. Therefore, by combining both techniques, it is possible to achieve a more significant improvement in performance, enabling the model to better adapt to cross-subject EEG emotion recognition tasks. However, effectively coordinating domain adaptation and fine-tuning while capitalizing on the strengths of each, reducing domain disparities, enhancing model adaptability, and ultimately improving accuracy is a challenging endeavor.

In order to address the aforementioned challenging task, a hybrid transfer learning strategy for cross-subject EEG emotion recognition is proposed. Specifically, Domain Adaptation with a Few-shot Fine-tuning Network (DFF-Net) is employed for cross-subject EEG emotion recognition. Firstly, the original EEG signals are divided into segments, each lasting 4 seconds. For each segment, Differential Entropy (DE) features in the δ, θ, α, β, and γ frequency bands are extracted. These features are spatially mapped based on electrode positions to generate EEG feature representations. To enhance the extraction of EEG features, a Vision Transformer (ViT) is employed as the Feature Extractor. Subsequently, building upon the original Domain-Adversarial Neural Network (DANN) model, a domain adaptive learning module named the Emo-DA module is devised for EEG emotion recognition. The module addresses domain discrepancies among different subjects. The Emo-DA module is then applied to pre-train a model on both the source and target domains. Fine-tuning is subsequently employed on the target domain to further test cross-subject EEG emotion recognition. Lastly, a series of comparative and ablation experiments are conducted using the DFF-Net framework. These experiments not only demonstrate the superiority of DFF-Net overall state-of-the-art models but also explore the contributions of key components within DFF-Net to the recognition performance in the cross-subject EEG emotion recognition task.

The primary contributions of this paper can be outlined as follows:

- With the aim of reducing the disparities between the source and target domains, a domain adaptive learning module for EEG emotion recognition was crafted, named the Emo-DA module. This module facilitates the model in achieving improved generalization on the target domain.
- In order to enhance the adaptability of the model to the target domain and make use of a limited amount of target domain data, the integration of domain adaptation and fine-tuning techniques is designed, leading to the creation of the domain adaptation with a few-shot fine-tuning network (DFF-Net). This approach is devised to better accommodate the specific features of the target domain, thereby enhancing the accuracy of the cross-subject EEG emotion recognition task.
- The proposed DFF-Net model achieves accuracy rates of 93.37 and 82.32% on the SEED and SEED-IV datasets, respectively, for cross-subject EEG emotion recognition. These rates surpass those of all state-of-the-art models. Furthermore, a series of ablation experiments were conducted to investigate the contributions of key components within DFF-Net to the recognition performance of cross-subject EEG emotion recognition tasks.

## 1 Related work

This section offers a thorough overview of relevant research in EEG-based emotion recognition, focusing on the application of transfer learning strategies in the context of cross-subject emotion recognition.

### 1.1 EEG-based emotion recognition

In recent years, there has been a notable proliferation of applications for electroencephalogram signals in the realm of emotion recognition. This heightened interest is primarily attributable to their inherent capacity to accurately and faithfully capture authentic emotional states within individuals. Prior investigations have predominantly concentrated on the enhancement of EEG-based emotion recognition methodologies through processes such as signal denoising, feature engineering, and classifier training. For instance, Wang et al. (2011) introduced an emotion recognition framework grounded in cerebral signals. This method integrated EEG spectral features with a Support Vector Machine (SVM) classifier, yielding experimental evidence that validates the viability of this strategy for precise emotion classification. Bahari and Janghorbani (2013) employed a non-linear method, specifically recurrence plot analysis, to extract distinctive features. These extracted features were subsequently utilized in combination with a K-Nearest Neighbors (KNN) classifier for the purpose of emotion recognition (Bahari and Janghorbani, 2013). However, traditional machine learning techniques are limited by the requirement of extensive feature engineering and feature selection, which often requires domain expertise.

In order to tackle the stated constraints, deep learning methodologies have been employed (Jia et al., 2022a,b). Given the ongoing enhancement and notable achievements of deep learning algorithms, EEG-based emotion recognition approaches utilizing deep learning frameworks have been successfully implemented, yielding encouraging outcomes. For example, Chen et al. (2019) introduced a deep CNN model inspired by those commonly used for image classification tasks in computer vision. This approach avoided the laborious task of manually extracting features and selecting attributes that conventional machine learning methods necessitate. Consequently, the precision and consistency of identifying emotions from EEG signals were substantially enhanced (Chen et al., 2019). Kwon et al. (2018) utilized Convolutional Neural Networks (CNN) to extract features from EEG signals. In this model, the EEG signal undergoes preprocessing with wavelet transform to consider both the temporal and frequency information before the convolution process (Kwon et al., 2018). Aside from the effective utilization of 2D Convolutional Neural Networks (2D-CNN) in EEG emotion recognition assignments, notable advancements have been achieved using 3D Convolutional Neural Networks (3D-CNN) as well. For instance, Salama et al. (2018) carried out an investigation into the application of 3D-CNN in the realm of emotion recognition. Additionally, they advanced a data augmentation phase to enhance the effectiveness of the 3D-CNN architectures (Salama et al., 2018). Moreover, Cho and Hwang (2020) introduced a 3D-CNN architecture that effectively captures the spatiotemporal portrayal of EEG signals to achieve precise emotion classification. In order to more effectively capture the global features of EEG signals, certain researchers have begun to explore the use of Transformer models for EEG emotion recognition. Liu et al. (2022) presented the EEG emotion Transformer (EeT) framework, which directly acquires spatial-spectral characteristics from EEG signal sequences, thereby modifying the conventional Transformer model for EEG data. Moreover, Wang et al. (2021b) put forward a model named Joint-Dimension-Aware Transformer (JDAT) for EEG emotion recognition. By applying adaptive compressed Multi-head Self-Attention (MSA) on multidimensional features, JDAT effectively focuses on various EEG information, encompassing spatial, frequency, and temporal domains (Wang et al., 2021b). Despite the successful applications of deep learning methods, the inherent diversity of human mental states, and varying responses to the same stimuli introduce challenges due to the non-stationary nature and individual variability of EEG signals (Jia et al., 2021b). Therefore, effectively modeling individual differences remains a challenge for the above-mentioned deep learning models in the context of cross-subject emotion recognition based on EEG signals. Nonetheless, transfer learning offers a promising strategy to address this issue.

### 1.2 Transfer learning for emotion recognition

Due to the potential applications of deep learning models in various domains, there has been significant interest in utilizing these models for EEG emotion recognition. However, when applying deep learning models to cross-subject EEG emotion recognition, the limited number of subjects in EEG emotion datasets, coupled with the inter-individual differences between subjects, presents a significant challenge. This often results in a notable performance decline for deep learning models in the context of cross-subject EEG emotion recognition tasks. To address the issue of performance degradation in EEG emotion recognition across subjects, many researchers have started exploring the application of transfer learning techniques. In cross-subject EEG emotion recognition tasks, transfer learning primarily addresses the issue of data domain gaps caused by individual differences. EEG signals from different subjects under the same emotional state can exhibit substantial variations due to individual differences. In this scenario, the target domain represents the feature space of EEG data obtained from a certain number of subjects, while the source domain encompasses the feature space of data collected from one or multiple different individuals. Fine-tuning, a widely used and effective knowledge transfer method in deep neural networks, has become a pivotal technology in the field of transfer learning. It facilitates adapting pre-trained models to specific tasks or domains. Fine-tuning enables the model to adjust its learned representations based on the characteristics of the target subjects, thereby enhancing the performance of cross-subject emotion recognition tasks. In order to investigate cross-subject emotion recognition through fine-tuning techniques, Li et al. (2018b) incorporate Fine-tuning into the emotion recognition networks and examine the extent to which the models can be shared among subjects. Wang et al. (2020) have proposed a method that utilizes fine-tuning to address the challenge of emotional differences across different datasets in deep model transfer learning, with the aim of constructing a robust emotion recognition model. Zhang et al. (2023) proposed a Self-Training Maximum Classifier Discrepancy (SMCD) framework for emotion recognition. This method entails the utilization of the fine-tuning strategy by implementing the model previously trained on the source domain onto the target domain. However, the above fine-tuning methods require a large amount of labeled data in the target domain for model refinement. If the labeled data in the target domain is limited, utilizing fine-tuning may become challenging. Therefore, some researchers have begun exploring the application of domain adaptation for cross-subject eeg emotion recognition. For example, Jin et al. (2017) introduced the implementation of the Domain Adaptation Network (DAN) for knowledge migration in EEG-based sentiment identification to tackle the core issue of reducing disparities between the origin participant and destination participant in an attempt to eradicate subject variability. Li et al. (2019) introduced a domain adaptation technique for EEG emotion recognition, enhanced by reducing the classification error on the source domain while simultaneously harmonizing the latent representations of the source and target domains to enhance their similarity. Wang et al. (2021a) introduced a proficient domain adaptation approach using the multi-subject learning paradigm to address cross-subject emotion classification tasks with restricted EEG data. This technique empowers the model to grasp overarching attributes from diverse participants and swiftly adjust to the specific target individual (Wang et al., 2021a). However, only relying on domain adaptation methods may not effectively leverage the available label information in the target domain, which can result in performance degradation. Domain adaptation typically focuses on reducing the performance gap between different domains, especially between the source and target domains. Fine-tuning involves further training on target domain data to adapt to its specific characteristics, thus enhancing the performance of the model in the target domain. Therefore, combining these two techniques can lead to more significant performance improvements, allowing the model to better adapt to cross-subject EEG emotion recognition tasks. However, effectively coordinating both domain adaptation and fine-tuning techniques and harnessing their strengths to reduce inter-domain differences, enhance model adaptability, and ultimately improve emotion recognition accuracy is a challenging task. The challenge of this task lies in the need for careful balance between the two techniques, ensuring that the model can adapt well to the data characteristics of the target domain while preserving its generalization capability. Additionally, the selection of appropriate domain adaptation methods and fine-tuning strategies must also be considered.

## 2 Preliminaries

In order to facilitate subsequent reading, some key content has been defined here:

Definition 1: The features $E=\left(E_{1},E_{2},...,E_{B}\right)\in {\mathbb{R}}^{N_{e}\times B}$ encompass *B* frequency bands derived from EEG signals, where *N*_*e*_ denotes the electrode count. The features $A^{E}=\left(A_{1}^{E},A_{2}^{E},...,A_{B}^{E}\right)\in {\mathbb{R}}^{\text{height and width of the feature map}\times B}$ are constructed, with *H* and *W* denoting the height and width of the feature map, respectively. This study aims to establish a correlation between these representations and emotional states. Given the representation *A*^*E*^, the task of emotion recognition can be represented as $Y_{out}=F\left(A^{E}\right)$, with *Y*_*out*_ signifying the emotional state and *F* representing the proposed model.

Definition 2: A labeled source domain is defined as $D_{s}^{E}={\left(A_{i}^{E},L_{i}^{E}\right)}_{i=1}^{N_{s}}$, and an unlabeled target domain is defined as $D_{t}^{E}={\left(A_{j}^{E}\right)}_{j=1}^{N_{t}}$. The joint probability distributions of the two domains are different, indicating that $P_{s}^{E}\left(A_{i}^{s},L_{i}^{s}\right)-P_{t}^{E}\left(A_{j}^{t},L_{j}^{t}\right)\neq 0$.

Definition 3: The unified representation of transfer learning methods can be expressed as Formula (1).


(1) $$\begin{array}{l} F^{*}=\underset{F}{\arg\min}\left(\frac{1}{N_{s}}\overset{N_{s}}{\underset{i=1}{\sum }}L_{s}\left(F\left(A_{i}^{s},Y_{i}^{s}\right)\right)+\lambda L_{t}\left(F\left(A_{j}^{t},Y_{j}^{t}\right)\right)+\alpha D\left(P_{s}^{E},P_{t}^{E}\right)\right) \end{array}$$

where F^*^ represents the optimized model parameters, *N*_*s*_ denotes the number of samples in the source domain, $F\left(A_{i}^{s},L_{i}^{s}\right)$ represents the prediction of the model *F* on the labeled samples from the source domain, $L_{s}$ denotes the loss function for the source domain, $F\left(A_{j}^{t},L_{j}^{t}\right)$ represents the prediction of the model *F* on the unlabeled samples from the target domain, $L_{t}$ represents the loss function for the target domain, λ and α are regularization parameters, $D\left(P_{s}^{E},P_{t}^{E}\right)$ is a discrepancy metric that measures the difference between the source and target domain distributions $P_{s}^{E}$ and $P_{t}^{E}$.

## 3 Methodology

### 3.1 Overview

**Figure 1:**
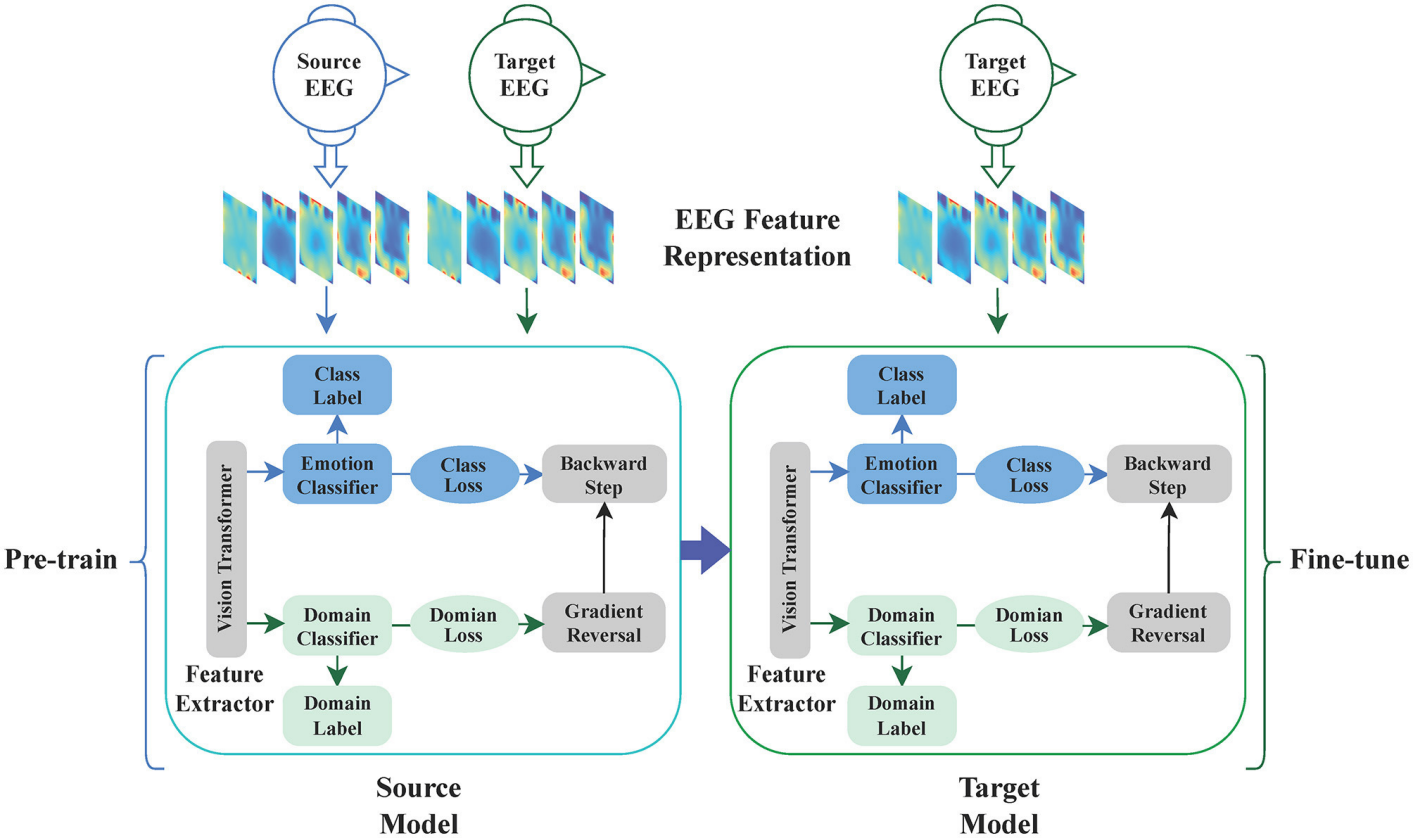
The whole process of EEG cross-subject emotion recognition. The entire process of EEG cross-subject emotion recognition begins with utilizing the EEG feature representation as input. Subsequently, the backbone model is employed for feature extraction. Next, the domain adaptation block is utilized to train a pre-trained model. Finally, this pre-trained model is fine-tuned for the task at hand.

Figure 1 illustrates the overall architecture of our proposed method, which consists of three main components: the backbone, domain adaptation, and fine-tuning. The first part is the backbone, which is a Vision Transformer primarily composed of a linear embedding layer and a Transformer encoder. The second part is domain adaptation, consisting of the backbone model, a domain classifier, a label predictor, domain loss, class loss, and gradient reversal. The third part is fine-tuning, involving pre-training, and fine-tuning. The method operates as follows: Initially, one model is trained by utilizing both source EEG data and target EEG data for the backbone and domain adaptation. After training, the model is saved and used as a pre-trained model. Finally, the target EEG data is divided into a few training samples and test samples, and then the pre-trained model is fine-tuned using the target data. Algorithm 1 shows the pseudocode for Domain Adaptation with Few-shot Fine-tuning. Initially, the Domain Adaptive Learning module for EEG emotion recognition (Emo-DA) is employed, taking labeled source domain data and unlabeled target domain data as input for Emo-DA. Subsequently, Fine-tuning is applied, utilizing a small amount of data from the target domain for training. Testing is then performed on the target domain, ultimately yielding the classification results.

**Algorithm 1:**
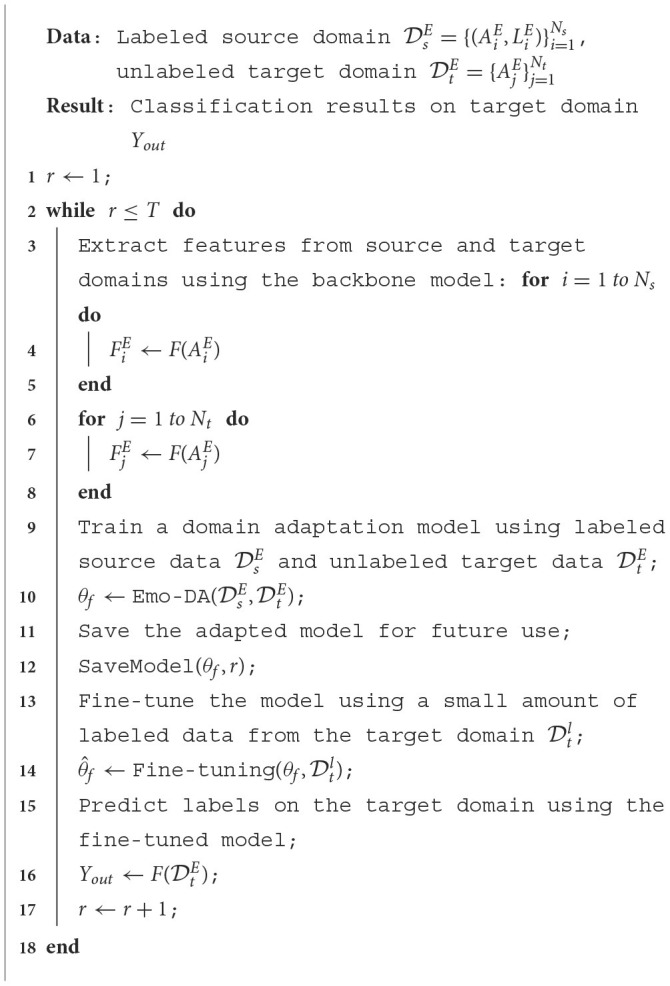
Domain adaptation with a few-shot fine-tuning.

### 3.2 EEG feature representations

**Figure 2:**
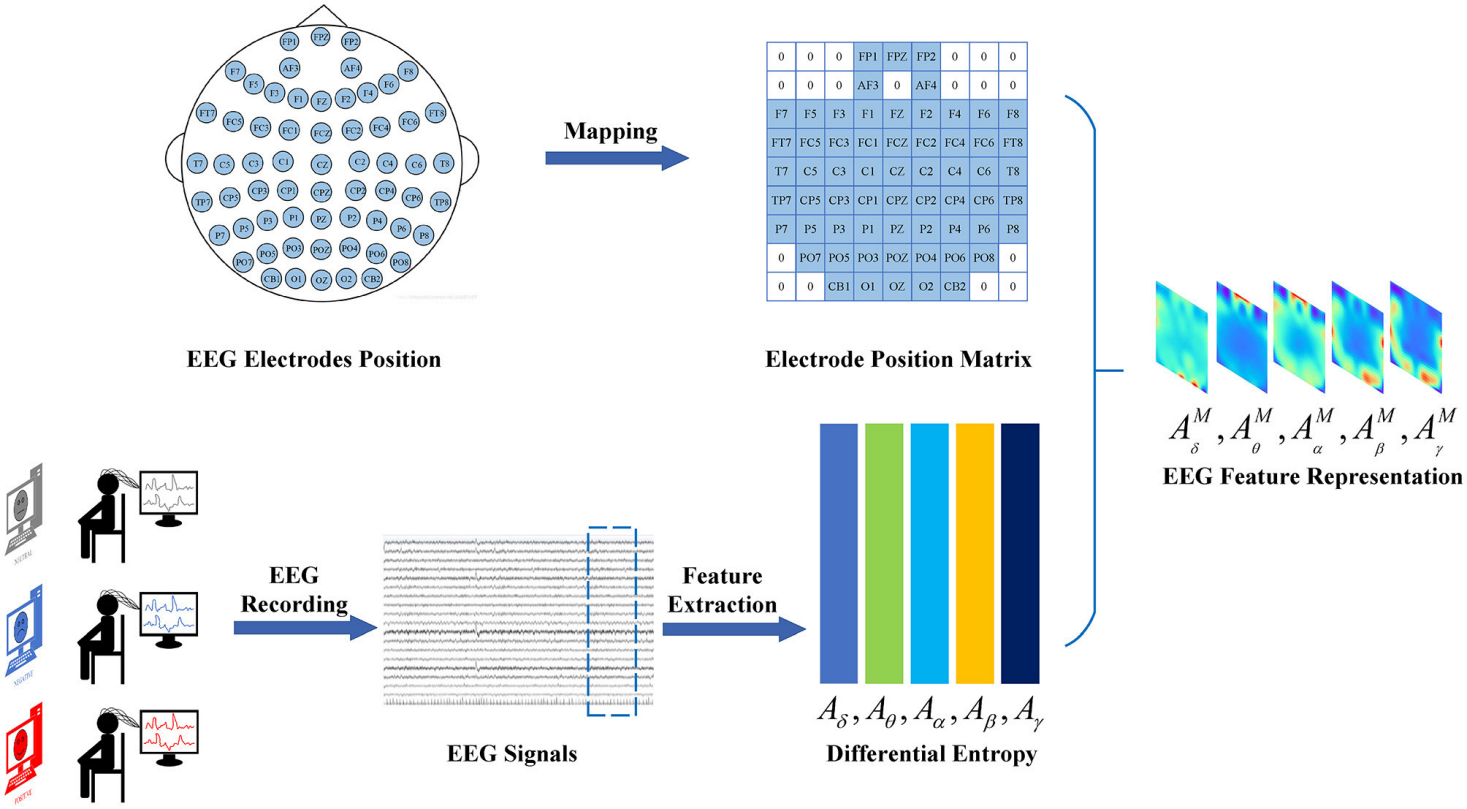
The transformation of the initial EEG signal into a feature representation. The EEG signal transformation process involves several steps. Firstly, the raw EEG signal is divided into segments of a fixed length. Subsequently, features in the frequency domain are extracted from each segment across various frequency bands. Finally, these features are then linked to the matrix representing the electrode positions, forming the EEG feature representations.

Figure 2 portrays the conversion process from the original EEG signals to EEG feature representations. The initial EEG signals are partitioned into non-overlapping 4-second intervals, maintaining identical labels as the original EEG signals. To form the EEG feature depiction, a method for extracting temporal-frequency features is employed, capturing the Differential Entropy (DE) attributes of five frequency ranges {δ, θ, α, β, γ} from the 4-second EEG segments across all EEG channels. The frequency feature $E=\left(E_{1},E_{2},...,E_{B}\right)\in {\mathbb{R}}^{N_{e}\times B}$ contains the extracted frequency bands from the DE feature. Here, *B* belongs to the set *B*∈{δ, θ, α, β, γ}, indicating the frequency band. *N*_*e*_ pertains to the electrodes, and is represented by *FP*1, *FPZ*, ..., *CB*2. The collection of EEG signals from all *N*_*e*_ electrodes on frequency band *B* is denoted as $S_{b}^{B}=\left(s_{b}^{1},s_{b}^{2},...,s_{b}^{N}\right)\in {\mathbb{R}}^{N}\left(b\in \left\{1,2,...,B\right\}\right)$. Subsequently, the chosen data undergo a transformation to generate a frequency-domain brain electrode location matrix denoted as $A_{b}^{M}\in {\mathbb{R}}^{H\times W}$. Here, *b*∈1, 2, ..., *B* represents the frequency band, *H* signifies the height of the matrix, and *W* indicates the width of the matrix. This transformation is performed based on the spatial arrangement of the electrodes on the brain. Finally, the EEG feature representation is crafted by combining frequency-domain brain electrode position matrices across various frequencies, resulting in $A^{M}=\left(A_{1}^{M},A_{2}^{M},...,A_{B}^{M}\right)\in {\mathbb{R}}^{H\times W\times B}$. This step accomplishes the construction of the EEG feature representation.

### 3.3 Transformer

**Figure 3:**
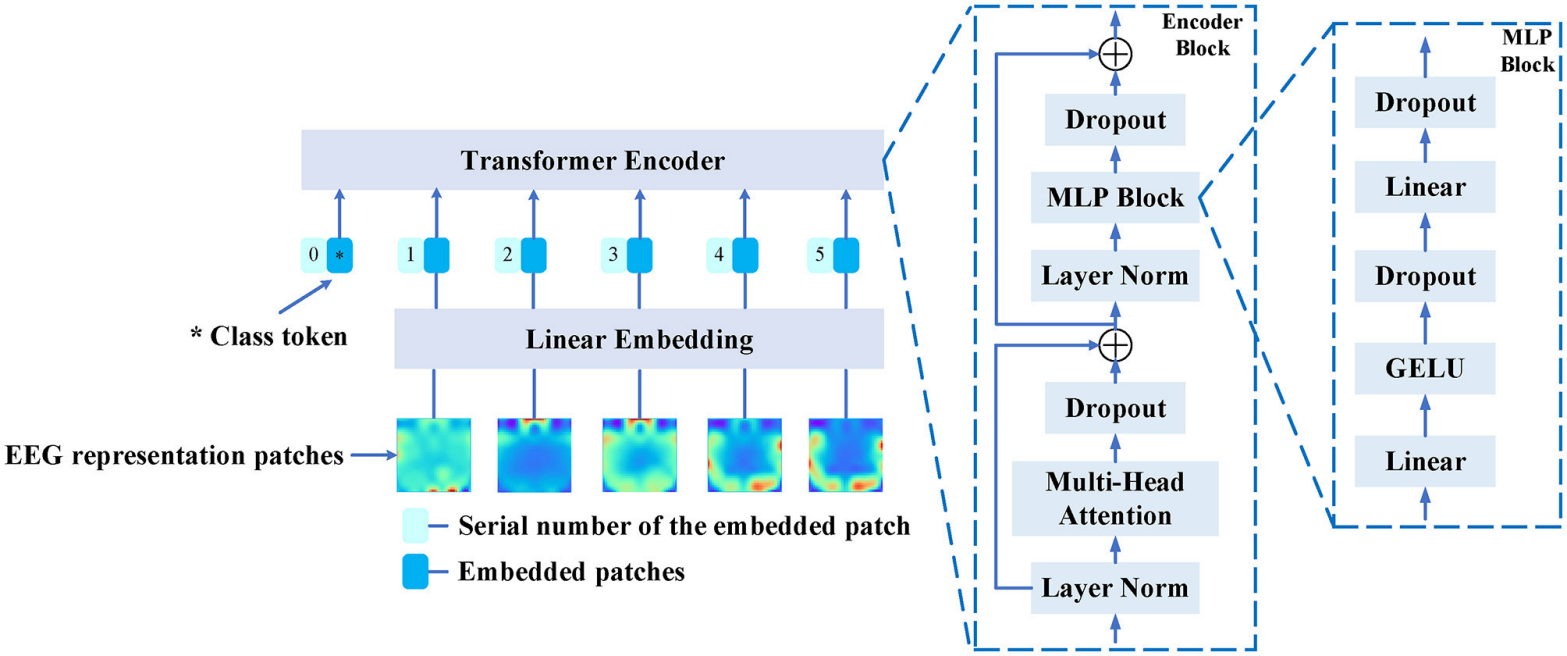
The structure of the Vision Transformer serves as the backbone for feature extraction from EEG feature representations. The backbone model takes the original EEG feature representation and divides it into EEG representation patches. Each representation patch is then used as input to the Linear Embedding layer. The output of the Linear Embedding layer serves as the input to the Transformer Encoder, which is composed of three main components: Multi-Head Attention, Multi-Layer Perceptron (MLP) Block, and Layer Normalization.

The Vision Transformer (ViT) has achieved state-of-the-art performance in various computer vision applications, such as image classification and segmentation. This motivates us to use ViT as the feature extractor for feature extraction from EEG feature representations. The architecture of the ViT as the feature extractor for feature extraction from EEG feature representations is illustrated in Figure 3.

The ViT model takes the EEG feature representation denoted as $A^{M}=\left(A_{1}^{M},A_{2}^{M},...,A_{B}^{M}\right)\in {\mathbb{R}}^{H\times W\times B}$ as input. This initial EEG feature representation, with dimensions *H* × *W* × *B*, is partitioned into *B* EEG representation patches $A_{b}^{M}\in {\mathbb{R}}^{H\times W}$, each patch having dimensions *H*×*W*. The representation patches are subsequently inputted into the Linear Embedding layer, which maps them to a fixed size denoted as *E*_*d*_. According to Equation (2), *W*_*A*_ can be deduced as the input for the Transformer Encoder. In this context, the class token, denoted by $x_{p}^{cls}\in {\mathbb{R}}^{E_{d}}$, contributes to feature representation learning. The parameter *N*_*TB*_∈*T, B* signifies the count of EEG representation patches, and $E_{A}\in {\mathbb{R}}^{H\times W\times E_{d}}$ represents the linear projection matrix. Additionally, the one-dimensional position embedding, $A_{E}^{pos}\in {\mathbb{R}}^{\left(N_{TB}+1\right)\times E_{d}}$, is introduced to maintain the sequential frequency series information.


(2) $$\begin{array}{l} W_{A}=\left[x_{p}^{cls};x_{p}^{1}E_{A};x_{p}^{2}E_{A};...;x_{p}^{N_{TB}}E_{A}\right]+A_{E}^{pos} \end{array}$$

As shown in Figure 3, the Transformer Encoder block consists of three main components: Multi-Head Attention, Multi-Layer Perceptron (MLP) block, and Layer Normalization. The Multi-Head Attention component initially performs self-attention computations using a multi-head mechanism. Each vector of position simultaneously considers information from other positions to capture global correlations. The output from the Multi-Head Attention is fed into a feedforward neural network in the MLP Block. Typically, the MLP includes two fully connected layers and a Gaussian Error Linear Unit (GELU) activation function, introducing non-linear transformations to further adjust and enrich feature representations. The output from the MLP block then undergoes layer normalization, which normalizes the feature vectors to reduce internal covariate shift, thereby enhancing model training stability and generalization capability. Once these three steps are completed, the output of the Transformer Encoder block becomes the input for the next layer. This process is repeated multiple times, typically involving multiple Encoder blocks stacked together. This way, the Transformer progressively extracts and transforms features from the input, resulting in gradually abstracted feature representations.

### 3.4 Domain adaptation network

**Figure 4:**
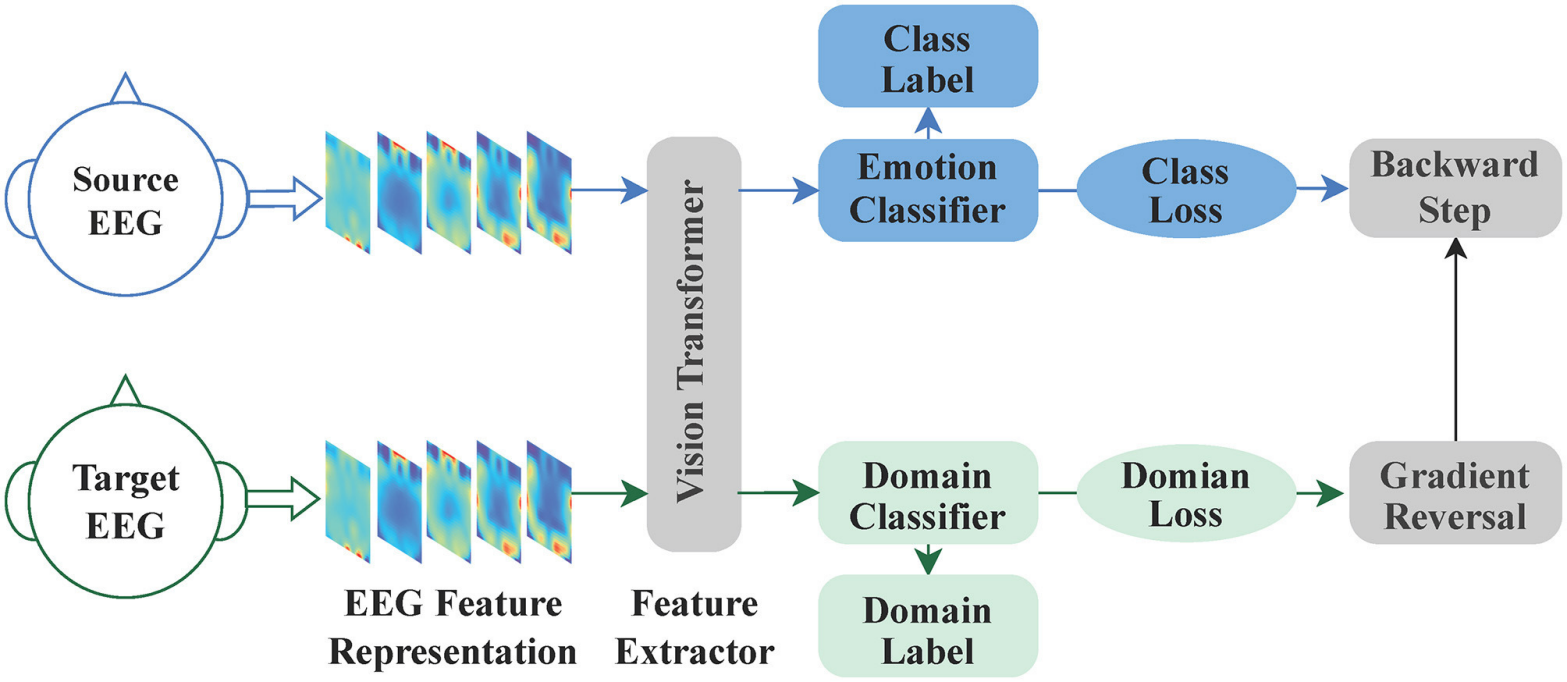
The Emo-DA module is an emotion recognition network consisting of three main components: a feature extractor, an emotion classifier, and a domain classifier. The feature extractor and emotion classifier work together to form a conventional feedforward neural network, which can be utilized for label prediction. Domain adaptation is achieved by employing the feature extractor and domain classifier in conjunction with backpropagation. Specifically, the gradient flowing from the domain classifier to the feature extractor is multiplied by negative parameters in the gradient reversal layer. This process ensures domain-invariant feature learning and facilitates the alignment of the source and target domains.

A Domain-Adversarial Neural Network (DANN) is utilized for achieving transfer learning. This framework was originally proposed by Ganin and Lempitsky (2015) for image classification. Based on the original DANN model, a domain adaptive learning module for EEG emotion recognition is proposed, named the Emo-DA module, with the aim of addressing domain discrepancies among different subjects. The primary objective of the Emo-DA module is to learn feature representations that have strong generalization capabilities, enabling the alignment of emotional data from different subjects in the shared feature space. The architecture of the Emo-DA module comprises three main components: a feature extractor, an emotion classifier, and a domain classifier, as illustrated in Figure 4.

The Feature Extractor is used to extract shared EEG emotion representations from both the source and target domain input data. For the Emo-DA module, the Vision Transformer model is selected as the Feature Extractor. The formula for the Feature Extractor in the Emo-DA module can be represented as Equation (3):


(3) $$\begin{array}{l} H_{i}=F_{\theta }\left(x_{i};\theta_{f}\right) \end{array}$$

where *x*_*i*_ represents the input sample, and *H*_*i*_ represents the output feature representation from the Feature Extractor. The Feature Extractor maps the input sample *x*_*i*_ to a high-level feature space using the parameter θ_*f*_, which contains abstract features useful for the emotion recognition task. These features will be passed to the Emotion Classifier and Domain Classifier for subsequent emotion recognition and domain adaptive learning.

The Emotion Classifier is a classifier used for emotion classification. It takes the shared features extracted by the Feature Extractor as input and performs emotion classification on the source domain data. In this case, a fully connected layer is chosen as the classifier for emotion classification. The formula for the Emotion Classifier in the Emo-DA module can be represented as Equation (4):


(4) $$\begin{array}{l} Y_{i}=G_{\phi }\left(H_{i};\phi_{y}\right) \end{array}$$

where *H*_*i*_ represents the output feature representation from the Feature Extractor, and *Y*_*i*_ represents the emotion prediction results of the model for the input sample *x*_*i*_. The Emotion Classifier maps the feature representation *H*_*i*_ to a predicted probability distribution over emotion labels using the parameter ϕ_*y*_.

The Domain Classifier is used to differentiate the features between the source and target domains. It takes the shared features extracted by the Feature Extractor as input and attempts to correctly classify them as belonging to either the source or target domain. The objective of the Domain Classifier is achieved through adversarial training, which aims to make the extracted features indistinguishable with respect to the domain. The formula for the Domain Classifier in the Emo-DA module can be represented as Equation (5):


(5) $$\begin{array}{l} D_{i}=D_{\psi }\left(H_{i};\psi_{d}\right) \end{array}$$

where *H*_*i*_ represents the output feature representation from the Feature Extractor, and *D*_*i*_ represents the prediction results of the domain label for the input sample *x*_*i*_. The Domain Classifier maps the feature representation *H*_*i*_ to a predicted probability distribution over domain labels using the parameter ψ_*d*_.

The Emo-DA module is capable of learning generalizable feature representations from the emotion data of different subjects, resulting in improved emotion recognition performance on both the source and target domains. Through domain adaptive training, the Emo-DA module aligns the feature representations of the source and target domains, thereby enhancing the generalization ability and adaptability of the model to the target domain. The overall training objective of the Emo-DA module can be expressed as Equation (6).


(6) $$\begin{array}{l} E\left(\theta_{f},\phi_{y},\psi_{d}\right)=\underset{x_{i}\in D_{s}^{E}}{\sum }L_{emotion}\left(G_{\phi }\left(F_{\theta }\left(x_{i}\right)\right),L_{i}^{y}\right)-\lambda \\ \underset{x_{i}\in D_{s}^{E}\cup D_{t}^{E}}{\sum }L_{domain}\left(D_{\psi }\left(F_{\theta }\left(x_{i}\right)\right),L_{i}^{d}\right) \end{array}$$

where θ_*f*_, ϕ_*y*_, and ψ_*d*_ represent the parameters of the feature extractor *F*_θ_, the emotion classifier *G*_ϕ_, and the domain classifier *D*_ψ_, respectively. $L_{emotion}$ denotes the emotion classification loss, while $L_{domain}$ represents the domain classification loss. The emotion samples are denoted by *x*_*i*_, and $L_{i}^{y}$ represents their corresponding true emotion labels. Additionally, $L_{i}^{d}$ represents their corresponding domain labels, where $L_{i}^{d}=0$ indicates that the sample *x*_*i*_ comes from the source domain, and $L_{i}^{d}=1$ indicates that the sample *x*_*i*_ comes from the target domain. The Emo-DA module first optimizes the parameters θ_*f*_ and ϕ_*y*_ of the feature extractor *F*_θ_ and emotion classifier *G*_ϕ_ by minimizing the classification loss and the feature extractor loss. This is achieved through the following formula, as shown in Equation (7):


(7) $$\begin{array}{l} \left({\hat{\theta }}_{f},{\hat{\phi }}_{y}\right)=\underset{\theta_{f},\phi_{y}}{\arg\min}E\left(\theta_{f},\phi_{y},\psi_{d}\right) \end{array}$$

Then, The Emo-DA module optimizes the parameters ψ*d* of the Domain Classifier *Dψ* by maximizing its loss. This is achieved through the following formula, as shown in Equation (8):


(8) $$\begin{array}{l} \left({\hat{\psi }}_{d}\right)=\underset{\psi_{d}}{\arg\max}E\left(\theta_{f},\phi_{y},\psi_{d}\right) \end{array}$$

The two steps mentioned above are alternated until the network converges. During the domain adaptive learning process, a Gradient Reversal Layer is employed to induce the feature extractor to learn adversarial feature representations, as shown in Equation (9):


(9) $$\begin{array}{l} {\tilde{x}}_{i}=R_{\lambda }\left(x_{i};\lambda \right) \end{array}$$

where *x*_*i*_ represents the input feature representation, *R*_λ_ is the function of the Gradient Reversal Layer, and λ is a hyper-parameter that controls the strength of gradient reversal. During the forward, the Gradient Reversal Layer behaves as an identity map, making ${\tilde{x}}_{i}$ and *x*_*i*_ equal. However, during the backward, the gradient of ${\tilde{x}}_{i}$ is reversed by multiplying it with −λ, effectively reversing the gradient direction. The Gradient Reversal Layer aims to minimize the loss for the emotion classification task and maximize the loss for the domain classification task in order to align the feature representations of the source and target domains. By doing so, the feature extractor can learn to effectively reduce the impact of domain differences, aligning the representations between the source and target domains, thereby mitigating the influence of domain discrepancies.

### 3.5 Pre-training and fine-tuning

Pre-training and Fine-tuning are model-based transfer learning methods. The primary objective of this approach is to identify shared parameter information between the source and target domains, facilitating knowledge transfer. When provided with a target domain dataset $D_{t}^{E}$, Pre-training and Fine-tuning leverage prior knowledge θ_*s*_ to learn a function represented by the parameters θ, as illustrated in Equation (10).


(10) $$\begin{array}{l} \theta^{*}=\underset{\theta }{\arg\min}L\left(\theta |\theta_{s},D_{t}^{E}\right) \end{array}$$

where θ^*^ represents the optimized model parameters, and $L\left(\theta |\theta_{s},D_{t}^{E}\right)$ is the loss function that measures the discrepancy between the model predictions using the parameters θ and the target domain data $D_{t}^{E}$, considering the prior knowledge θ_*s*_. The optimization aims to find the best parameters θ that minimize the loss function and facilitate effective knowledge transfer from the source domain to the target domain.

**Figure 5:**
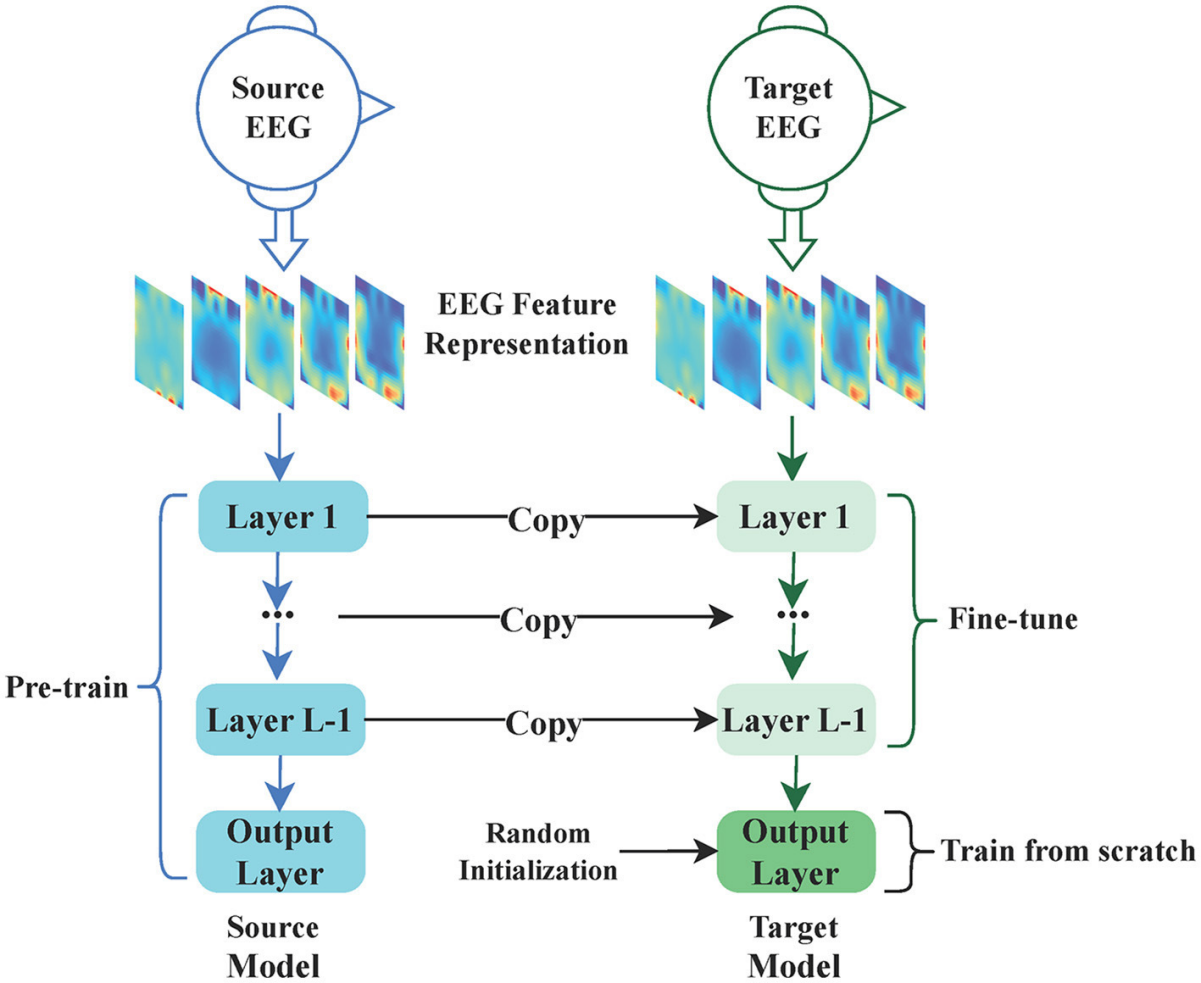
The Pre-training and Fine-tuning process for EEG emotion classification. This process involves two main components: Pre-training and Fine-tuning. In the Pre-training phase, the source EEG feature representation serves as the input to the source model, and upon completion of training, the source model is saved. During Fine-tuning, the target EEG feature representation is utilized as the input for the target model. The target model is created based on the pre-trained source model, with certain layer parameters being fixed, while only the remaining layers undergo fine-tuning.

Figure 5 illustrates a straightforward Pre-training and Fine-tuning process for EEG emotion classification. As shown in the figure, the source EEG feature representation is used as input for Pre-training the source model, and the model is saved after Pre-training. The target EEG feature representation is then utilized as input for Fine-tuning the target model. Subsequently, the source model is adapted by fixing the parameters of the early layers obtained from Pre-training and Fine-tuning the subsequent layers specifically for the EEG emotion classification task. Finally, the target model is constructed. This approach not only significantly accelerates the network training speed but also greatly improves the performance of the EEG emotion classification task.

## 4 Experiments

In this section, the introduction of two widely used datasets and the description of the experiment settings are presented. Subsequently, a comparison is made between our proposed method and the baseline method. Finally, ablation experiments are conducted, and the results are reported and discussed.

### 4.1 Datasets

The study was carried out using the SEED (Zheng and Lu, 2015) and SEED-IV (Zheng et al., 2018) datasets. Both are public EEG datasets used primarily for emotion recognition. The SEED dataset consists of a total of 62 channels of EEG signals recorded from 15 subjects who participated in 15 experiments. During the experiments, the subjects were presented with 15 Chinese film clips. The clip-viewing process was divided into four stages, including a 5-second start prompt, a 4-min clip period, a 45-second self-assessment, and a 15-second rest period. The researchers categorized the movie clips into three emotional categories: negative, neutral, and positive. The positive movies were comedies intended to evoke feelings of happiness, while the negative movies were tragic films meant to elicit feelings of sadness. The neutral movies were world heritage documentaries designed not to evoke either positive or negative emotions.

The SEED-IV dataset is an expansion of the SEED dataset, encompassing 72 meticulously chosen film clips. These clips were carefully selected to elicit emotions of happiness, sadness, fear, or neutrality in the viewers. The primary objective of these video clips is to evoke corresponding emotions in the subjects participating in the experiment. Following the viewing of the video clips, the subjects self-assessed their emotions. The experiment involved a total of 15 subjects, with each participant participating in 3 sessions on different days. Each session consisted of 24 trials, where the participant watched one of the film clips.

The EEG signals from both the SEED datasets and SEED-IV datasets were recorded using the ESI Neuroscan system, with 62 channels and a sampling rate of 1,000 Hz. Subsequently, the signals were downsampled to 200 Hz. To enhance the data quality, a band-pass filter was applied to remove noise and artifacts. Following the preprocessing step, various features, including Differential Entropy, were extracted from each segment in five frequency bands: δ (1–4 Hz), θ (4–8 Hz), α (8–14 Hz), β (14–31 Hz), and γ (31–50 Hz). Table 1 provides a summary of the processing steps conducted to extract the EEG data from the SEED and SEED-IV datasets.

**Table 1:** The processing involved in extracting the SEED and SEED-IV datasets are summarized.

	**SEED**	**SEED-IV**
Number of electrodes	62	62
Number of video clips	15	24
Number of sessions	3	3
Number of subjects	15	15
Type of emotion	Positive	Happy
	Neutral	Sad
	Negative	Fear
		Neutral
Frequency band	γ: 31~50 Hz	γ:31~50 Hz
	β:14~31 Hz	β:14~31 Hz
	α:8~14 Hz	α:8~14 Hz
	θ:4~8 Hz	θ:4~8 Hz
	δ:1~4 Hz	δ:1~4 Hz
Bandpass frequency filter	0~75 HZ	1~75 HZ
Sampling rate	200 HZ	200 HZ

The differential entropy (DE) features stand out as the most pertinent EEG characteristics for emotion recognition (Zheng and Lu, 2015; Zheng et al., 2018; Hwang et al., 2020). Differential entropy acts as a continuum of Shannon entropy for continuous variables, quantifying the uncertainty inherent in the probability distribution of such continuous random variables. The initial formulation for DE is expressed through Formula (11).


(11) $$\begin{array}{l} \text{DE}=-\overset{\infty }{\underset{-\infty }{\int }}f\left(x\right)\ln\;\left(f\left(x\right)\right)dx \end{array}$$

where DE indicates the value of differential entropy, serving as a measure of the unpredictability associated with continuous random variables. For EEG signal analysis, it is assumed that the signal follows a normal distribution, specifically *x*~*N*(μ, σ^2^). To simplify the computation of the DE feature, Formula (12) is utilized. Formula (12) is given as follows.


(12) $$\begin{array}{lll} \text{DE} & = & -\overset{\infty }{\underset{-\infty }{\int }}\frac{1}{\sqrt{2\pi \sigma }}\exp\frac{{\left(x-\mu \right)}^{2}}{2\sigma^{2}}\ln\;\left(\frac{1}{\sqrt{2\pi \sigma }}\exp\frac{{\left(x-\mu \right)}^{2}}{2\sigma^{2}}\right)dx\\ = & \frac{1}{2}\ln\;2\pi e\sigma^{2} \end{array}$$

### 4.2 Experiments setting

In the experiments, the training and testing were performed on a Tesla V100-SXM2-32GB GPU, with the implementation carried out using the PyTorch framework. The main focus of the experiments was on cross-subject transfer. The EEG data of one subject was taken as the target domain, and the EEG data of all the remaining subjects served as the source domain. For training in the source domain, the source model was obtained. The samples in the source domain were randomly shuffled, and the data were divided into training and testing sets with a ratio of 7:3. Similarly, for training in the target domain, the target model was obtained. However, in this case, only 0.1% of the target domain samples were used for fine-tuning training, while the remaining samples were used for testing. During the training in both the source and target domains, the cross-entropy loss function was used. The summary of hyper-parameter settings for the experiments on the SEED and SEED-IV datasets is provided in Table 2.

**Table 2:** The summary of hyper-parameter settings for the experiments on the SEED and SEED-IV datasets.

	**SEED**	**SEED-IV**
Number of classes	3	4
Batch size	32	32
Optimizer	Adam	Adam
Learning rate	1e-5	1e-5
Loss function	Cross-entropy	Cross-entropy
Dropout rate	0.2	0.2
Encoder layer	12	12

### 4.3 Baseline methods

In order to evaluate the effectiveness of the proposed model, a comparative analysis was conducted with several baseline methods using the SEED and SEED IV datasets. Brief introductions to each of these methods are provided below.

- DDC (Tzeng et al., 2014): The suggested domain adaptation strategy utilizes the utmost mean discrepancy (MMD) to diminish the total distribution discrepancy between the source and target domains. It combines an adjustment stratum and a domain perplexity loss, both grounded on MMD, to aid the spontaneous attainment of a mutual portrayal, concurrently refining categorization effectiveness, and confirming domain constancy.
- DCORAL (Sun and Saenko, 2016): An unsupervised approach for domain adaptation is utilized to attain complete end-to-end adaptation within deep neural networks. The primary aim is to mitigate the divergence in statistical characteristics of the source and target feature activations.
- DAN (Li et al., 2018a): The suggested Domain Adaptation Network (DAN) utilizes the combined reduction of multi-kernel Maximum Mean Discrepancies (MK-MMDs) and loss specific to the task. This allows the DAN to adeptly tackle distinctions between different domains while retaining features pertinent to the task.
- SOGNN (Li et al., 2021): A Self-Organized Graph Neural Network (SOGNN) is introduced for the purpose of cross-subject EEG emotion recognition. The graph framework of the SOGNN is autonomously built employing a self-organized module for every signal.
- MS-MDA (Chen et al., 2021a): Multi-Source Marginal Distribution Adaptation (MS-MDA) is utilized to address multiple sources, each having distinct features. This method involves generating separate branches by pairing each source domain with the target domain, facilitating one-to-one Domain Adaptation.
- MEERNet (Chen et al., 2021b): A network for recognizing emotions based on EEG signals from various sources (MEERNet) is introduced. MEERNet is structured with a shared feature extractor, domain-specific feature extractors, and domain-specific classifiers. Through harnessing insights from diverse source domains, the model adeptly conveys insights to the intended domain.
- MSMRA (Cao et al., 2022): The technique of multi-origin and multi-presentation adjustment (MSMRA) is applied for emotion recognition in EEG across different domains. This entails segmenting EEG data stemming from varied participants into numerous fields and harmonizing the distribution of assorted representations acquired from a blended framework. Moreover, this strategy introduces a feature extraction module specialized for multiple domains, aiming to extract numerous elevated-level characteristics of varying dimensions.
- SDDA (Li et al., 2022): The method introduced is termed Semi-supervised Dynamic Domain Adaptation (SDDA). Within SDDA, a limited set of labeled instances from the target domain is utilized to assess and enhance the Label-specific Domain (LSD) characteristics. Furthermore, cross-entropy (CE) is applied as the classification loss on source data that are sampled independently. Through the simultaneous minimization of the Global Domain Discrepancy (GDD), LSD, and CE, the model proficiently acquires intricate attributes for emotion recognition in situations involving varying subjects.
- DSAAN (Meng et al., 2022): Presents the Deep Subdomain Associate Adaptation Network (DSAAN), an approach for EEG emotion recognition utilizing transfer learning. Domains are subdivided based on sample labels, where genuine labels are utilized for the source domain, and forecasted pseudo-labels are applied to the target domain. DSAAN operates as a transfer network, harmonizing subdomain distributions through the Subdomain Associate Loop (SAL). Adaptation is accomplished via the minimization of a unified loss encompassing source domain classification and SAL.
- MS-ADA (She et al., 2023): An approach for identifying emotions that utilizes a multi-origin linked domain adaptation (DA) framework to integrate features that are both consistent across domains and distinctive to each domain.
- SMCD (Zhang et al., 2023): A Self-Training Maximum Classifier Disparity (SMCD) framework is utilized for emotion recognition across different individuals. This encompasses the utilization of the previously trained model on the source area in the target realm, which results in the establishment of feature clusters within the target realm. The method to forestall excessive knowledge adaptation from the existing source individuals involves the adjustment of the model through fine-tuning, utilizing a restricted count of annotated calibration samples from the novel individual.

### 4.4 Experimental results

Table 3 displays the cross-participant experimental results on the SEED and SEED-IV datasets, showcasing the mean accuracy (ACC), and dispersion (STD) of both the reference approaches and the proposed DFF-Net framework for emotion recognition based on EEG signals. Within the SEED dataset, the outcomes indicate our strategy surpasses alternative methodologies in the inter-subject transfer scenario, accomplishing a mean accuracy of 93.37% alongside a dispersion of 1.88%. Concerning the SEED-IV dataset, encompassing a four-category classification assignment, the performance of the technique is relatively lower in contrast to the SEED dataset. Specifically, for the SEED-IV dataset, our technique attains an average accuracy of 82.3%, coupled with a corresponding dispersion of 5.38%.

**Table 3:** Comparing the performance of baseline methods with the proposed DFF-Net on the SEED and SEED-IV datasets.

**Method**	**Year**	**SEED**		**SEED-IV**	
		**ACC (%)** ↑	**STD (%)** ↓	**ACC (%)** ↑	**STD (%)** ↓
DDC (Tzeng et al., 2014)	2014	68.99	3.23	37.41	6.36
DCORAL (Sun and Saenko, 2016)	2016	62.14	7.98	40.50	10.05
DAN (Li et al., 2018a)	2018	83.81	8.56	58.87	8.13
SOGNN (Li et al., 2021)	2021	86.81	5.79	75.27	8.19
MS-MDA (Chen et al., 2021a)	2021	79.67	8.01	57.92	10.12
MEERNet (Chen et al., 2021b)	2021	87.1	2.0	71.0	12.1
MSMRA (Cao et al., 2022)	2022	87.62	7.53	69.77	7.37
SDDA (Li et al., 2022)	2022	91.08	7.70	81.58	8.72
DSAAN (Meng et al., 2022)	2022	89.23	1.93	-	-
MS-ADA (She et al., 2023)	2023	86.16	7.87	59.29	13.65
SMCD (Zhang et al., 2023)	2023	88.75	8.68	74.49	13.80
**DFF-Net**	**2023**	**93.37**	**1.88**	**82.32**	**5.38**

DDC introduced a domain confusion loss to AlexNet and fine-tuned it on both the source and target domains. Similarly, DAN shares similarities with DDC but employs a multi-kernel selection technique for improved average embedding alignment and multi-layer adaptation. As a result, Our proposed approach demonstrates significantly higher accuracy compared to traditional methods such as DDC, DAN, and DCORAL. When compared to the best-performing method among these traditional approaches, namely DAN, our method exhibits an accuracy increase of 9.56% on the SEED dataset and a remarkable 23.45% increase on the SEED IV dataset. Compared to methods such as MS-MDA, MS-ADA, and SDDA that solely employ domain adaptation techniques, our proposed approach effectively harmonizes cross-domain features through fine-tuning, mitigating domain shifts, and promoting domain-invariant representations. In comparison to the best-performing method among these, SDDA, Our method effectively harmonizes domain adaptation and fine-tuning, leveraging the individual strengths of both domain adaptation and fine-tuning to reduce domain discrepancies and enhance model adaptability. This results in a remarkable 2.29% accuracy improvement on the SEED dataset and a notable 0.74% accuracy increase on the SEED IV dataset.

**Figure 6:**
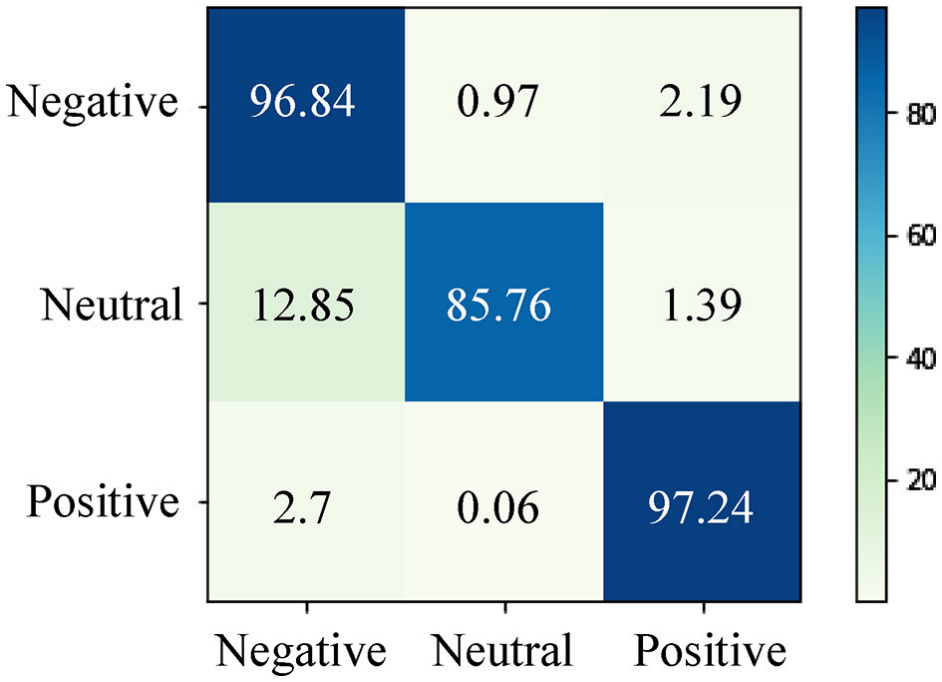
The confusion matrix for EEG emotion recognition in the cross-subject task on the SEED dataset. Our proposed method demonstrates effective classification for positive and negative emotions, while its performance is comparatively weaker for neutral emotions.

Figure 6 presents the confusion matrix depicting the predictions made by our proposed method for EEG emotion recognition in the cross-subject task on the SEED dataset. It is evident from Figure 6 that our proposed approach performs well in accurately classifying positive and negative emotions. However, its performance appears comparatively weaker in the case of neutral emotions. This observation can be attributed to several factors. Firstly, positive and negative emotions tend to be more intense and elicit stronger neural responses compared to neutral emotions. This heightened intensity can result in more distinct and easily detectable features within EEG data, which are more amenable to classification. Secondly, the challenges in recognizing neutral emotions could stem from variations in neural responses exhibited by different subjects toward neutral stimuli. This diversity of responses among subjects makes it more challenging for the model to accurately classify neutral emotions in a cross-subject scenario.

**Figure 7:**
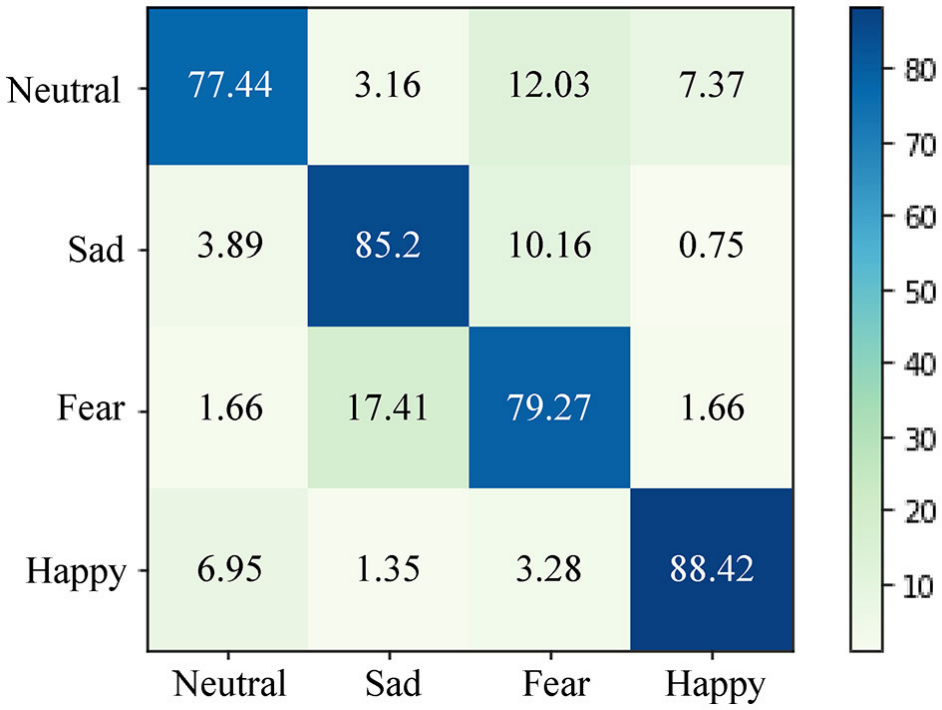
The confusion matrix for EEG emotion recognition in the cross-subject task on the SEED IV dataset. Our proposed method excels in classifying happy and sad emotions effectively. However, its performance is relatively weaker in distinguishing neutral and fearful emotions.

Figure 7 depicts the confusion matrix of predictions generated by our proposed approach for EEG emotion recognition in the cross-subject task on the SEED IV dataset. As the SEED IV dataset involves four-class classification, the overall performance of the model on the SEED IV dataset is comparatively lower than that on the SEED dataset. From Figure 7, it is evident that our proposed method excels in classifying happy and sad emotions. However, its performance in classifying neutral and fearful emotions is relatively weaker. This phenomenon can be attributed to the complexity of neutral emotions, characterized by subtle and less pronounced neural patterns. Individuals might exhibit diverse neural responses to neutral stimuli, posing challenges to the consistent classification of the model across different subjects. Similarly, fear emotions can manifest various neural responses based on personal experiences, potentially increasing the difficulty of achieving accurate cross-subject classification.

### 4.5 Ablation experiments

**Figure 8:**
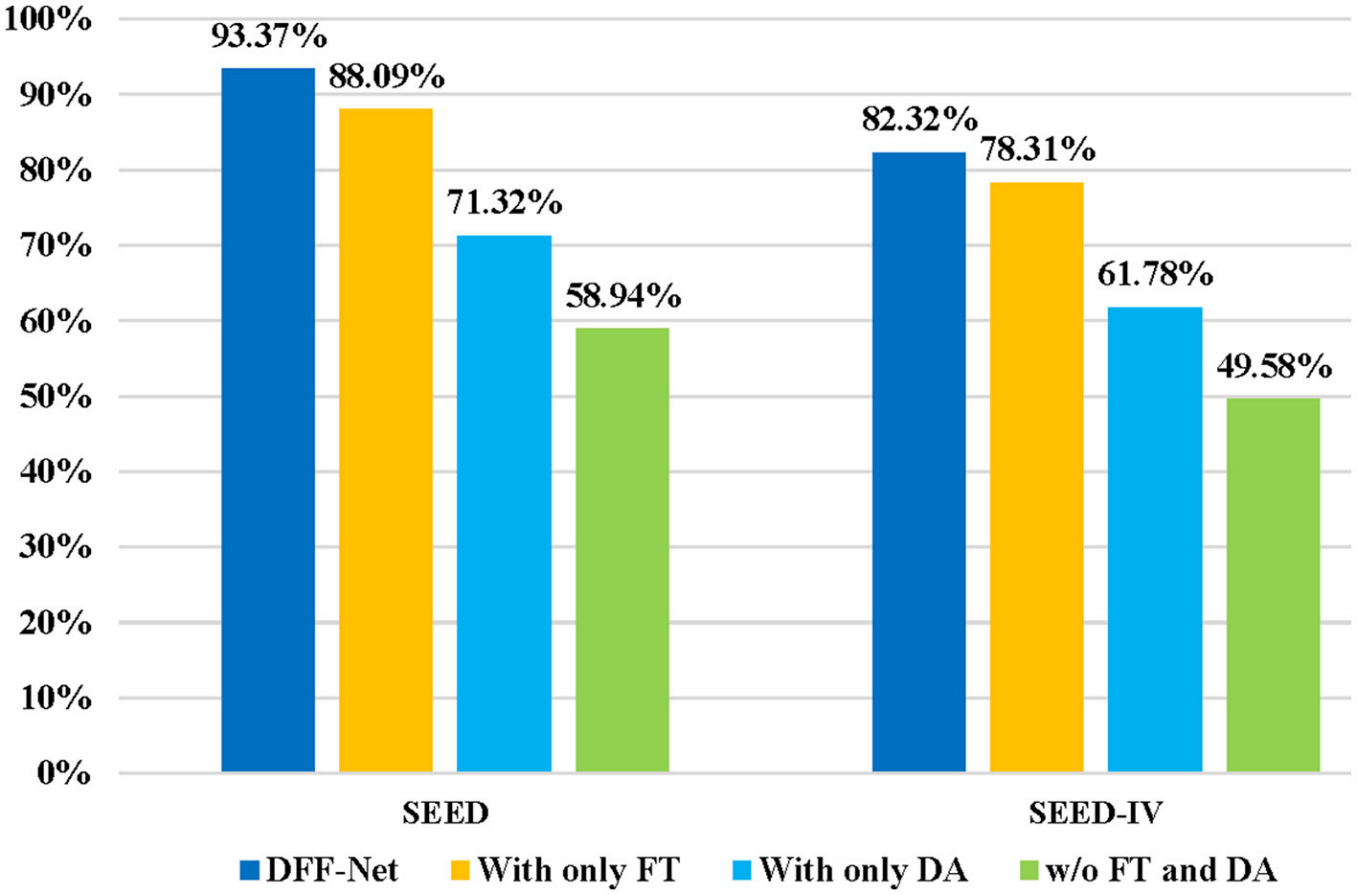
Ablation experiments on the major components of DFF-Net. The accuracy of “w/o FT and DA” on the SEED dataset is 58.94%, and it drops to 49.58% on the SEED-IV dataset. In contrast, “With only DA” demonstrates improved results, achieving 71.32% on SEED and 61.78% on SEED-IV, respectively. Worth noting is that “With only FT” outperforms the others, achieving 88.09% on SEED and 78.31% on SEED-IV. Our comprehensive DFF-Net model showcases superior performance, achieving significant accuracy rates of 93.37% on SEED and 82.32% on SEED-IV, surpassing all other methods, thus validating the effectiveness of the proposed approach.

In order to validate the effects of different components in our model on the EEG emotion recognition tasks, we performed ablation experiments on both the SEED and SEED IV datasets. Our proposed method, termed DFF-Net, primarily consists of two components: Domain Adaptation and Fine-tuning. To validate the effectiveness of these two key components in our approach, we conducted ablation experiments on DFF-Net. Figure 8 illustrates the influence of these two key components of DFF-Net on the cross-subject EEG emotion recognition task. “With only FT” indicates the utilization of Fine-tuning alone for the purpose of cross-subject EEG emotion recognition, which implies the exclusion of the Emo-DA module. In this case, the model is trained on the source domain and then fine-tuned on the target domain for subsequent testing in cross-subject EEG emotion recognition. “With only DA” indicates utilizing Domain Adaptation solely for cross-subject EEG emotion recognition. This entails performing Domain Adaptation on both the source and target domains and directly testing cross-subject EEG emotion recognition. “w/o FT and DA” implies the absence of both Fine-tuning and Domain Adaptation methods. In this scenario, the model trained on the source domain is directly tested on the target domain for cross-subject EEG emotion recognition.

“With only FT” achieved an accuracy rate of 88.09% on the SEED dataset and 78.31% on the SEED IV dataset. In contrast, “With only DA” achieved an accuracy of 71.32% on the SEED dataset and 61.78% on the SEED IV dataset. This indicates that the Fine-tuning method performs better in the cross-subject EEG emotion recognition task compared to the Domain Adaptation approach. Additionally, the performance of “With only FT” on both the SEED and SEED IV datasets is lower than that of the DFF-Net. This further validates the effectiveness of the proposed Emo-DA module. The accuracy of “w/o FT and DA” on the SEED dataset is 58.94%, while on the SEED IV dataset, it is 49.58%. This suggests that the generalization ability of the ViT model is limited, and without the utilization of transfer learning methods, achieving the cross-subject EEG emotion recognition task is challenging. DFF-Net achieved an accuracy of 93.37% on the SEED dataset and 83.32% on the SEED IV dataset, both surpassing the results of other methods in the ablation experiments. These outcomes collectively indicate that the combination of Fine-tuning and Domain Adaptation contributes to enhancing the recognition performance of the model in the cross-subject EEG emotion recognition task.

**Figure 9:**
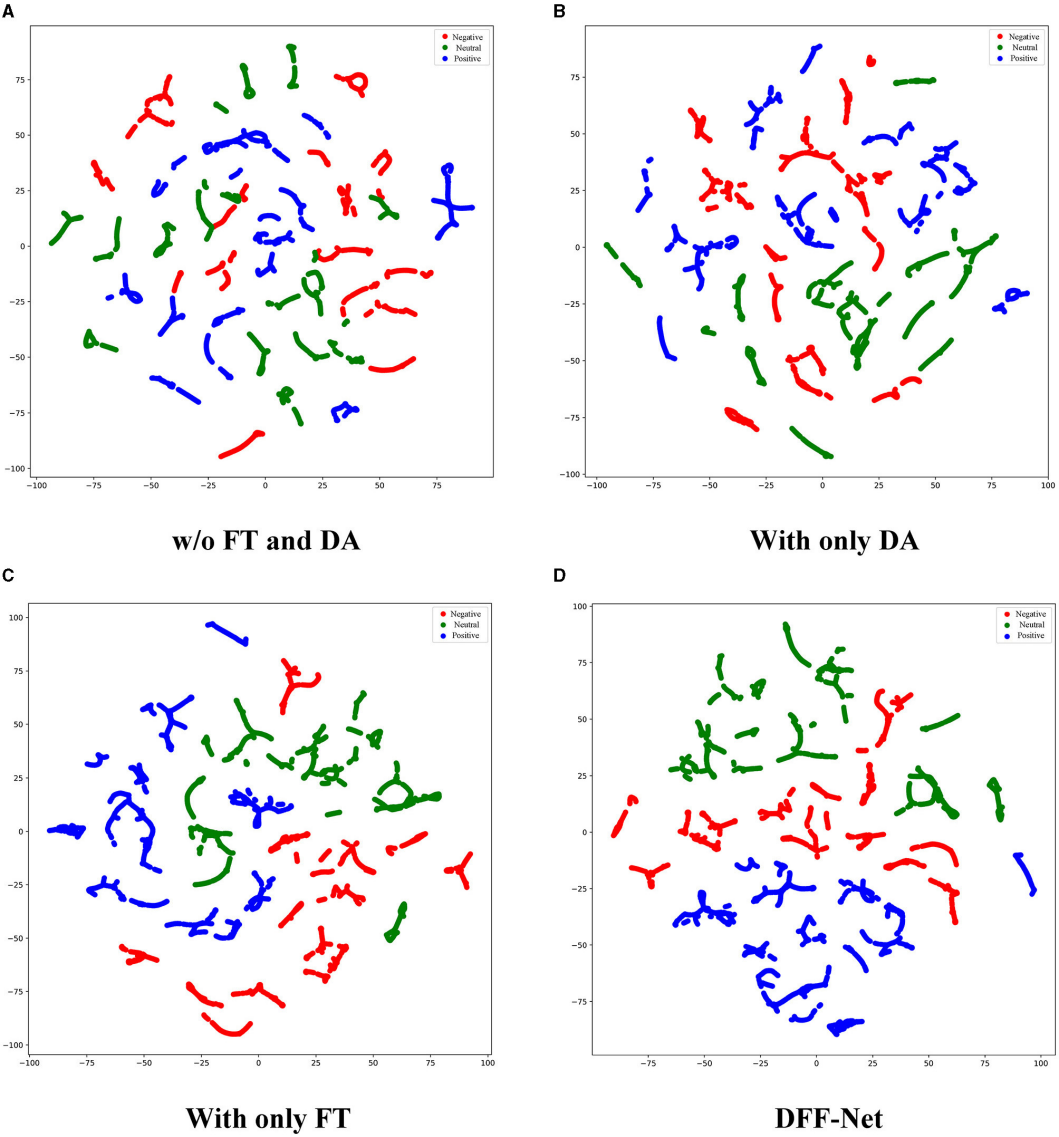
The performance of various methods in cross-subject EEG emotion recognition tasks was visualized using t-SNE. For simplicity, we only use the SEED dataset as an example. Data points are color-coded to represent three different emotions: negative (red), neutral (green), and positive (blue). **(A)** This figure shows the scatter plot without using any fine-tuning or domain adaptation methods. **(B)** This figure displays the scatter plot using only domain adaptation. **(C)** This figure represents the scatter plot with only fine-tuning methods applied. **(D)** This figure illustrates the scatter plot using DFF-Net.

To intuitively comprehend the effectiveness of the DFF-Net, We randomly selected a subject from the SEED dataset and used their EEG samples as the test set. The data was visualized using a scatter plot with t-SNE (Van der Maaten and Hinton, 2008), as shown in Figure 9. More precisely, we selected four methods for visualization experiments: “w/o FT and DA,” “With only DA,” “With only FT,” and “DFF-Net.” Data points are color-coded to represent three different emotions: negative is denoted by red, neutral by green, and positive by blue. It's worth noting that the data range after dimensionality reduction varies for different subjects. Here, we only showcase the visualization results of our method. The figure displays scatter plots for four distinct methods. As depicted in Figure 9A, data points corresponding to the three emotions are significantly intertwined, exhibiting pronounced overlap. This suggests that “w/o FT and DA” might face challenges in differentiating emotions in cross-subject EEG emotion recognition tasks. As seen in Figure 9B, clusters appear somewhat separated, but there remains considerable overlap between emotions, particularly between the negative and neutral states. This indicates that while “With only DA” shows improvement over “w/o FT and DA,” it might not be adequate by itself for optimal emotion recognition. In Figure 9C, the clustering of each emotion appears more pronounced compared to both “w/o FT and DA” and “With only DA.” This implies that “With only FT” significantly enhances the distinguishability of emotions. As illustrated in Figure 9D, the clusters for each emotion are distinctly different and well-separated, especially the positive (blue) cluster, which is almost entirely isolated from the other two emotions. This further indicates that the combination of fine-tuning and domain adaptation contributes to enhanced recognition performance in cross-subject EEG emotion recognition tasks.

### 4.6 Discussion and analysis

This paper presents a hybrid transfer learning strategy for cross-subject EEG emotion recognition, aiming to overcome the limitations of using a single transfer learning strategy in this task. In contrast to other single-transfer learning strategies, the proposed hybrid transfer learning strategy involves an analysis of two common transfer learning methods: domain adaptation and fine-tuning. By effectively integrating the distinctive characteristics of domain adaptation and fine-tuning at the methodological level, the domain adaptation with a few-Shot fine-tuning network (DFF-Net) is proposed, creating a novel hybrid transfer learning strategy. The results of DFF-Net on the SEED and SEED IV datasets demonstrate its superior performance in cross-subject EEG emotion recognition compared to other state-of-the-art methods. This introduces a fresh strategy for future EEG-based emotion recognition systems. Several noteworthy discussion points arise from the proposed DFF-Net model.

The performance disparity of our proposed DFF-Net model between the SEED dataset and the SEED IV dataset is quite noticeable. Under identical experimental settings, the accuracy achieved in SEED IV dataset experiments is lower than that in SEED dataset experiments. The reasons for this discrepancy can be delineated as follows: Firstly, the SEED-IV dataset comprises four emotion classes: Happy, Sad, Fear, and Neutral, while the SEED dataset has three classes: Positive, Neutral, and Negative. Consequently, the experimental complexity of the SEED IV dataset is significantly higher compared to the SEED dataset. Secondly, the feature extractor of the DFF-Net model employs a Transformer architecture, a type of model that typically performs better with larger sample sizes. Given that the SEED IV dataset contains fewer samples than the SEED dataset, the performance of the model on the SEED IV dataset is comparatively inferior. Lastly, variations in sample quality between the SEED dataset and the SEED IV dataset contribute to the divergent performance of the DFF-Net model on these two datasets.

To validate the significance of the Emo-DA module and fine-tuning components within our proposed DFF-Net model, we conducted ablation experiments on the DFF-Net model. The experiments were carried out under the same experimental settings. From the experimental results, it is evident that the strategy of disregarding any form of transfer learning yields the poorest outcomes. This strategy involves testing the transformer model directly on the target domain after training on the source domain. This further underscores the limitations of deep learning models in cross-subject EEG emotion recognition.

Another observation from the experimental results is that employing the fine-tuning strategy alone surpasses the performance of using domain adaptation alone. This can be attributed to several factors. Firstly, fine-tuning enables the model to adjust its parameters according to the specifics of the target domain. This flexibility aids the model in maintaining alignment with subtle variations in target domain data, thereby enhancing its performance in cross-subject EEG emotion recognition. Secondly, domain adaptation cannot completely mitigate all domain discrepancies, such as variations in electrode placement or signal noise, which significantly impact model performance. Fine-tuning assists the model in more accurately learning these domain-specific features.

In the final experimental results, it becomes evident that the performance of our proposed DFF-Net model surpasses that of using the fine-tuning strategy alone. This is because DFF-Net, as a hybrid transfer learning strategy, benefits from the Emo-DA module, which facilitates more effective alignment between the source and target domain distributions. In contrast to the standalone fine-tuning strategy, DFF-Net better leverages valuable information from the source domain while adjusting according to specific features of the target domain. In conclusion, the Hybrid Transfer Learning Strategy of DFF-Net effectively addresses the limitations of using domain adaptation or fine-tuning in isolation. This comprehensive approach ensures that DFF-Net can harness the advantages of domain adaptation and fine-tuning, consequently enhancing the accuracy of cross-subject EEG emotion recognition tasks.

## 5 Conclusion

In this paper, we introduce a hybrid transfer learning strategy, specifically referred to as the domain adaptation with a few-Shot fine-tuning Network (DFF-Net), for the task of cross-subject EEG emotion recognition. First, we extract Differential Entropy (DE) features and map them spatially based on the electrode positions to generate the EEG feature representation, which serves as the input for our proposed model. Then, we employ the Vision Transformer (ViT) as the Feature Extractor, and building upon the original Domain-Adversarial Neural Network (DANN) model, we develop a domain adaptive learning module for EEG emotion recognition, named the Emo-DA module. Finally, we apply the Emo-DA module to pre-train a model on both the source and target domains, and then use fine-tuning on the target domain for cross-subject EEG emotion recognition testing. This approach is designed to better adapt to the specific features of the target domain, thereby enhancing the accuracy of the cross-subject EEG emotion recognition task. The proposed DFF-Net achieved average recognition accuracies of 93.37% on the SEED dataset and 82.32% on the SEED-IV dataset, surpassing the state-of-the-art methods. To assess the impact of different components in DFF-Net on EEG emotion recognition tasks, we conducted ablation experiments on both the SEED and SEED-IV datasets. The experimental results demonstrate that the integration of domain adaptation and fine-tuning effectively enhances the adaptability of the model to the target domain, mitigates the influence of domain discrepancies, and minimizes the reliance on large annotated datasets in the target domain. Ultimately, this approach significantly improves the accuracy of cross-subject EEG emotion recognition. The proposed DFF-Net introduces a novel approach for cross-subject EEG emotion recognition tasks. This method can also be easily applied to other cross-subject EEG classification tasks, such as motor imagery and sleep stage classification. However, the current model still has some limitations in practical applications. For instance, the model lacks the capability for real-time online processing, and it requires a small number of samples from the target domain during training. In future work, we will investigate the real-time online capability and domain generalization of DFF-Net for cross-subject EEG emotion recognition, aiming to further enhance its model generalization and practicality.

## Data availability statement

Publicly available datasets were analyzed in this study. This data can be found here: https://bcmi.sjtu.edu.cn/home/seed/seed.html.

## Author contributions

WL: Conceptualization, Data curation, Formal analysis, Methodology, Resources, Software, Visualization, Writing – original draft, Writing – review & editing. HL: Formal analysis, Funding acquisition, Project administration, Writing – review & editing. HM: Conceptualization, Formal analysis, Funding acquisition, Project administration, Resources, Software, Supervision, Writing – review & editing. T–PT: Conceptualization, Data curation, Formal analysis, Funding acquisition, Investigation, Methodology, Project administration, Resources, Validation, Visualization, Writing – review & editing. LX: Project administration, Funding acquisition, Software, Writing – review & editing.

## References

[B1] Almarri B. Rajasekaran S. Huang C.-H. (2021). Automatic subject-specific spatiotemporal feature selection for subject-independent affective bci. PLoS ONE 16, e0253383. 10.1371/journal.pone.0253383 34437542 PMC8389489

[B2] Atkinson J. Campos D. (2016). Improving bci-based emotion recognition by combining EEG feature selection and kernel classifiers. Expert Syst. Appl. 47, 35–41. 10.1016/j.eswa.2015.10.049

[B3] Bahari F. Janghorbani A. (2013). “EEG-based emotion recognition using recurrence plot analysis and k nearest neighbor classifier,” in 2013 20th Iranian Conference on Biomedical Engineering (ICBME) 228–233. 10.1109/ICBME.2013.6782224

[B4] Cao J. He X. Yang C. Chen S. Li Z. Wang Z. (2022). Multi-source and multi-representation adaptation for cross-domain electroencephalography emotion recognition. Front. Psychol. 12, 809459. 10.3389/fpsyg.2021.809459 35095696 PMC8792438

[B5] Chen H. Jin M. Li Z. Fan C. Li J. He H. (2021a). Ms-mda: Multisource marginal distribution adaptation for cross-subject and cross-session EEG emotion recognition. Front. Neurosci. 15, 778488. 10.3389/fnins.2021.778488 34949983 PMC8688841

[B6] Chen H. Li Z. Jin M. Li J. (2021b). “Meernet: multi-source EEG-based emotion recognition network for generalization across subjects and sessions,” in 2021 43rd Annual International Conference of the IEEE Engineering in Medicine &Biology Society (EMBC) (IEEE), 6094–6097. 10.1109/EMBC46164.2021.9630277 34892507

[B7] Chen J. Zhang P. Mao Z. Huang Y. Jiang D. Zhang Y. (2019). Accurate EEG-based emotion recognition on combined features using deep convolutional neural networks. IEEE Access 7, 44317–44328. 10.1109/ACCESS.2019.2908285

[B8] Cho J. Hwang H. (2020). Spatio-temporal representation of an electoencephalogram for emotion recognition using a three-dimensional convolutional neural network. Sensors 20, 3491. 10.3390/s20123491 32575708 PMC7349167

[B9] Cimtay Y. Ekmekcioglu E. Caglar-Ozhan S. (2020). Cross-subject multimodal emotion recognition based on hybrid fusion. IEEE Access 8, 168865–168878. 10.1109/ACCESS.2020.3023871

[B10] Doma V. Pirouz M. (2020). A comparative analysis of machine learning methods for emotion recognition using EEG and peripheral physiological signals. J. Big Data 7, 1–21. 10.1186/s40537-020-00289-7

[B11] Ganin Y. Lempitsky V. (2015). “Unsupervised domain adaptation by backpropagation,” in International Conference on Machine Learning (PMLR), 1180–1189.

[B12] Huang Z. Ma Y. Wang R. Li W. Dai Y. (2023). A model for EEG-based emotion recognition: CNN-BI-LSTM with attention mechanism. Electronics 12, 3188. 10.3390/electronics12143188

[B13] Hwang S. Hong K. Son G. Byun H. (2020). Learning CNN features from de features for EEG-based emotion recognition. Patt. Anal. Appl. 23, 1323–1335. 10.1007/s10044-019-00860-w

[B14] Jia Z. Cai X. Jiao Z. (2022a). Multi-modal physiological signals based squeeze-and-excitation network with domain adversarial learning for sleep staging. IEEE Sensors J. 22, 3464–3471. 10.1109/JSEN.2022.3140383

[B15] Jia Z. Ji J. Zhou X. Zhou Y. (2022b). Hybrid spiking neural network for sleep electroencephalogram signals. Sci. China Inf. Sci. 65, 140403. 10.1007/s11432-021-3380-1

[B16] Jia Z. Lin Y. Cai X. Chen H. Gou H. Wang J. (2020). “SST-emotionnet: spatial-spectral-temporal based attention 3D dense network for EEG emotion recognition,” in Proceedings of the 28th ACM International Conference on Multimedia 2909–2917. 10.1145/3394171.3413724

[B17] Jia Z. Lin Y. Wang J. Feng Z. Xie X. Chen C. (2021a). “Hetemotionnet: two-stream heterogeneous graph recurrent neural network for multi-modal emotion recognition,” in Proceedings of the 29th ACM International Conference on Multimedia 1047–1056. 10.1145/3474085.3475583

[B18] Jia Z. Lin Y. Wang J. Ning X. He Y. Zhou R.. (2021b). Multi-view spatial-temporal graph convolutional networks with domain generalization for sleep stage classification. IEEE Trans. Neural Syst. Rehab. Eng. 29, 1977–1986. 10.1109/TNSRE.2021.3110665 34487495 PMC8556658

[B19] Jin Y.-M. Luo Y.-D. Zheng W.-L. Lu B.-L. (2017). “EEG-based emotion recognition using domain adaptation network,” in 2017 International Conference on Orange Technologies (ICOT) (IEEE), 222–225. 10.1109/ICOT.2017.8336126

[B20] Kwon Y.-H. Shin S.-B. Kim S.-D. (2018). Electroencephalography based fusion two-dimensional (2D)-convolution neural networks (CNN) model for emotion recognition system. Sensors 18, 1383. 10.3390/s18051383 29710869 PMC5982398

[B21] Li H. Jin Y.-M. Zheng W.-L. Lu B.-L. (2018a). “Cross-subject emotion recognition using deep adaptation networks,” in Neural Information Processing: 25th International Conference, ICONIP 2018, Siem Reap, Cambodia, December 13–16, 2018, Proceedings, Part V 25 (Springer), 403–413. 10.1007/978-3-030-04221-9_36

[B22] Li J. Li S. Pan J. Wang F. (2021). Cross-subject EEG emotion recognition with self-organized graph neural network. Front. Neurosci. 15, 611653. 10.3389/fnins.2021.611653 34177441 PMC8221183

[B23] Li J. Qiu S. Du C. Wang Y. He H. (2019). Domain adaptation for EEG emotion recognition based on latent representation similarity. IEEE Trans. Cogn. Dev. Syst. 12, 344–353. 10.1109/TCDS.2019.2949306

[B24] Li J. Zhang Z. He H. (2018b). Hierarchical convolutional neural networks for EEG-based emotion recognition. Cogn. Comput. 10, 368–380. 10.1007/s12559-017-9533-x

[B25] Li Z. Zhu E. Jin M. Fan C. He H. Cai T.. (2022). Dynamic domain adaptation for class-aware cross-subject and cross-session EEG emotion recognition. IEEE J. Biomed. Health Inform. 26, 5964–5973. 10.1109/JBHI.2022.3210158 36170411

[B26] Liu J. Wu H. Zhang L. Zhao Y. (2022). “Spatial-temporal transformers for EEG emotion recognition,” in 2022 The 6th International Conference on Advances in Artificial Intelligence 116–120. 10.1145/3571560.3571577

[B27] Meng M. Hu J. Gao Y. Kong W. Luo Z. (2022). A deep subdomain associate adaptation network for cross-session and cross-subject EEG emotion recognition. Biomed. Signal Proc. Control 78, 103873. 10.1016/j.bspc.2022.103873

[B28] Salama E. S. El-Khoribi R. A. Shoman M. E. Shalaby M. A. W. (2018). EEG-based emotion recognition using 3D convolutional neural networks. Int. J. Adv. Comput. Sci. Appl. 9, 843. 10.14569/IJACSA.2018.09084332845859

[B29] She Q. Zhang C. Fang F. Ma Y. Zhang Y. (2023). Multisource associate domain adaptation for cross-subject and cross-session EEG emotion recognition. IEEE Trans. Instrument. Measur. 72, 985. 10.1109/TIM.2023.3277985

[B30] Sun B. Saenko K. (2016). “Deep coral: Correlation alignment for deep domain adaptation,” in Computer Vision-ECCV 2016 Workshops: Amsterdam, The Netherlands, October 8–10 and 15–16, 2016, Proceedings, Part III 14 (Springer), 443–450. 10.1007/978-3-319-49409-8_35

[B31] Tan C. Ceballos G. Kasabov N. Puthanmadam Subramaniyam N. (2020). Fusionsense: emotion classification using feature fusion of multimodal data and deep learning in a brain-inspired spiking neural network. Sensors 20, 5328. 10.3390/s20185328 32957655 PMC7571195

[B32] Tzeng E. Hoffman J. Zhang N. Saenko K. Darrell T. (2014). Deep domain confusion: Maximizing for domain invariance. arXiv preprint arXiv:1412.3474.

[B33] Van der Maaten L. Hinton G. (2008). Visualizing data using t-SNE. J. Mach. Lear. Res. 9, 2579–2605.

[B34] Wang F. Wu S. Zhang W. Xu Z. Zhang Y. Wu C.. (2020). Emotion recognition with convolutional neural network and EEG-based efdms. Neuropsychologia 146, 107506. 10.1016/j.neuropsychologia.2020.107506 32497532

[B35] Wang X.-W. Nie D. Lu B.-L. (2011). “EEG-based emotion recognition using frequency domain features and support vector machines,” in Neural Information Processing, eds. Lu B.-L. Zhang L. Kwok J. (Berlin, Heidelberg: Springer), 734–743. 10.1007/978-3-642-24955-6_87

[B36] Wang Y. Liu J. Ruan Q. Wang S. Wang C. (2021a). Cross-subject EEG emotion classification based on few-label adversarial domain adaption. Expert Syst. Appl. 185, 115581. 10.1016/j.eswa.2021.115581

[B37] Wang Z. Chen M. Feng G. (2023). Study on driver cross-subject emotion recognition based on raw multi-channels EEG data. Electronics 12, 2359. 10.3390/electronics12112359

[B38] Wang Z. Zhou Z. Shen H. Xu Q. Huang K. (2021b). JDAT: Joint-dimension-aware transformer with strong flexibility for EEG emotion recognition. Technical Report. 10.36227/techrxiv.17056961

[B39] Xing X. Li Z. Xu T. Shu L. Hu B. Xu X. (2019). SAE+ LSTM: a new framework for emotion recognition from multi-channel EEG. Front. Neurorob. 13, 37. 10.3389/fnbot.2019.00037 31244638 PMC6581731

[B40] Zhang X. Huang D. Li H. Zhang Y. Xia Y. Liu J. (2023). Self-training maximum classifier discrepancy for EEG emotion recognition. CAAI Trans. Intell. Technol. 10.1049/cit2.12174

[B41] Zheng W.-L. Liu W. Lu Y. Lu B.-L. Cichocki A. (2018). Emotionmeter: a multimodal framework for recognizing human emotions. IEEE Trans. Cyber. 49, 1110–1122. 10.1109/TCYB.2018.2797176 29994384

[B42] Zheng W.-L. Lu B.-L. (2015). Investigating critical frequency bands and channels for EEG-based emotion recognition with deep neural networks. IEEE Trans. Auton. Mental Dev. 7, 162–175. 10.1109/TAMD.2015.2431497

[B43] Zhou X. Lin D. Jia Z. Xiao J. Liu C. Zhai L.. (2023a). An EEG channel selection framework for driver drowsiness detection via interpretability guidance. arXiv preprint arXiv:2304.14920. 10.1109/EMBC40787.2023.10341126 38083658

[B44] Zhou X. Liu C. Zhai L. Jia Z. Guan C. Liu Y. (2023b). Interpretable and robust AI in EEG systems: a survey. arXiv preprint arXiv:2304.10755.

